# Study method for a single-arm, multi-center implementation study of strategies for use by healthcare professionals in offering smokers an adapted proactive referral for online smoking cessation treatment in health check-up settings: a hybrid type 3 effectiveness-implementation study (N-EQUITY2405)

**DOI:** 10.3389/fpubh.2026.1843479

**Published:** 2026-07-15

**Authors:** Keiichi Yuwaki, Taichi Shimazu, Chie Taniguchi, Miyuki Odawara, Junko Saito, Aya Kuchiba, Manami Inoue, Hirohito Sone, Masakazu Nakamura, Kiminori Kato

**Affiliations:** 1 Division of Behavioral Sciences, National Cancer Center Institute for Cancer Control, National Cancer Center, Chuo-ku, Tokyo, Japan; 2 Department of Cancer Epidemiology, Graduate School of Medicine, The University of Tokyo, Bunkyo-ku, Tokyo, Japan; 3 College of Nursing, Aichi Medical University, Nagakute, Aichi, Japan; 4 Graduate School of Public Health, Teikyo University, Itabashi-ku, Tokyo, Japan; 5 Division of Biostatistical Research, National Cancer Center Institute for Cancer Control, National Cancer Center, Chuo-ku, Tokyo, Japan; 6 Division of Prevention, National Cancer Center Institute for Cancer Control, National Cancer Center, Chuo-ku, Tokyo, Japan; 7 Department of Hematology, Endocrinology and Metabolism, Niigata University Faculty of Medicine, Niigata, Japan; 8 Rural Health Research Center, Institute of Community Medicine, Japan Association for Development of Community Medicine, Chiyoda-ku, Tokyo, Japan; 9 Division of Population Data Science, National Cancer Center Institute for Cancer Control, National Cancer Center, Chuo-ku, Tokyo, Japan; 10 Department of Prevention of Noncommunicable Diseases and Promotion of Health Checkup, Graduate School of Medical and Dental Sciences, Niigata University, Niigata, Japan; 11 Niigata Association of Occupational Health, Niigata, Japan

**Keywords:** clinical trial protocol, implementation science, pragmatic clinical trial, referral and consultation, smoking cessation, tobacco

## Abstract

**Introduction:**

Proactive referral, defined as a strategy to connect smokers with smoking cessation programs under the responsibility of healthcare professionals, is effective in increasing the uptake of such programs. A feasibility study in a health check-up setting of the Brief Tobacco Intervention with Proactive Referral (BTI-PR), our smoker-level implementation strategy for smoking cessation treatment (SCT), revealed that the BTI-PR could be delivered to 85.7% of smokers as intended by healthcare professionals following provider-level guidance on implementation, which included educational sessions and the provision of materials. The present study aimed to estimate smoker uptake of SCT following a multifaceted, provider-level implementation strategy in Japanese real-world health check-up settings.

**Methods:**

This single-arm, multi-center, uncontrolled hybrid type 3 effectiveness-implementation study is focused on providing stakeholders with the evidence necessary to support adoption of the BTI-PR together with its provider-level implementation strategy. The BTI-PR is a smoker-level implementation strategy for SCT which acts by motivating smokers to schedule an online SCT appointment by themselves. The provider-level implementation strategy consists of eight components: (a) conduct a training session, (b) provide a guide on the BTI-PR, (c) provide a checksheet for the BTI-PR, (d) provide a flyer on the online SCT, (e) conduct a kickoff meeting and request to discuss pre-specifiable matters, (f) schedule a pre-implementation phase, (g) request holding a review meeting and report, and (h) conduct a plenary meeting to share local knowledge. Primary outcome is the penetration of online SCT, namely the proportion of smokers eligible for the BTI-PR who attend the first session of the online SCT within 3 months after receiving the BTI-PR.

**Results:**

Data collection was completed in February 2026. A total of 24 healthcare professionals who received the provider-level implementation strategy delivered the BTI-PR to smokers to explore the primary outcome. The final analyses were commenced in March 2026.

**Conclusion:**

This study is expected to support the adoption of proactive referral together with an implementation strategy in health check-up settings, although causal inference remains limited. The findings will contribute to the implementation of effective smoking cessation support into preventive health services.

**Trial registration number:**

https://center6.umin.ac.jp/cgi-open-bin/ctr_e/ctr_view.cgi?recptno=R000064502; UMIN-CTR, number UMIN000056447.

## Introduction

Tobacco smoking is a leading cause of multiple diseases, including cancer, cardiovascular disease, and chronic respiratory disease (1, 2). Smoking cessation prevents these diseases and premature death (3). The provision of behavioral support and pharmacotherapy by medical professionals aids smokers to achieve smoking cessation (4). In Japan, for example, approximately 80% of smokers using smoking cessation treatment (SCT) in clinics, which has been covered by the public health insurance system since 2006 (5), achieved four-week continuous abstinence at the end of a five-session treatment course delivered over a 12-week period (6, 7). A simulation study estimated that SCT enabled a large number of smokers to improve their health over their lifetime with no additional financial cost (8). However, this cost-effective SCT is used by only 1% of smokers annually (9). A similar evidence-practice gap in the use of smoking cessation programs among smokers has been observed worldwide (10, 11).

Proactive referral, defined as a strategy to connect smokers to smoking cessation programs, including SCT, under the responsibility of healthcare professionals, is effective in increasing the uptake of such programs (12). In many previous studies of proactive referral, however, the healthcare site, such as a clinic, reported the smoker’s contact information to a smoking cessation program, such as Quitline, whose staff then contacted the smoker (12, 13). We considered that if an appointment system for attendance at a smoking cessation program were established, smokers could schedule appointments with the program directly, with the assistance of healthcare site staff. This direct referral would relieve program staff of the time and responsibility of contacting smokers and would accordingly be preferred by them.

General health check-ups aimed at prevention and screening of diseases have been implemented in some countries, including Japan (14–16). Health check-ups represent a teachable moment, or in other words an opportune time for healthcare professionals to provide smoking cessation support (SCS) to smokers by communicating the risks of smoking and the benefits of quitting as a means of motivating them to use SCT (17, 18). A brief SCS has been reported to increase the abstinence rate by 3.29 times in comparison to usual care (19, 20). The brief SCS, a less intensive population-wide approach, can serve as a gateway to SCT, a more intensive individual approach (21). However, an evidence-practice gap exists in SCS delivery among healthcare professionals. A cross-sectional questionnaire survey revealed that only 27% of healthcare professionals working in health check-up settings informed smokers of the contact information for SCT through SCS (22). Furthermore, approximately 70% of these professionals perceived a lack of self-efficacy and knowledge as barriers to delivering SCS (22). Decreasing this evidence-practice gap in SCS delivery will require strategies to overcome such barriers.

We previously developed the Brief Tobacco Intervention with Proactive Referral (BTI-PR), a smoker-level implementation strategy which is aimed at increasing the use of SCT (23). The BTI-PR is an SCS strategy that incorporates a brief SCS into adapted proactive referral for use in health check-up settings in Japan. In the BTI-PR, smokers schedule the appointment themselves under the supervision of healthcare professionals. In an earlier feasibility study, healthcare professionals who had received a multifaceted provider-level implementation strategy to overcome barriers to SCS delivery, including educational sessions and provision of materials, could deliver the BTI-PR in the intended manner to 85.7% of eligible smokers (23). Furthermore, both healthcare professionals and smokers considered that the BTI-PR was acceptable and appropriate in health check-up settings (23).

The aim of the present study is to estimate smoker uptake of SCT following a multifaceted provider-level implementation strategy to promote delivery of the BTI-PR in real-world health check-up settings. This study is positioned as an implementation-focused effectiveness evaluation, which provides stakeholders with the evidence necessary to support the adoption of the BTI-PR together with its implementation strategy for healthcare professionals.

## Methods

### Overview of this study

**Figure 1:**
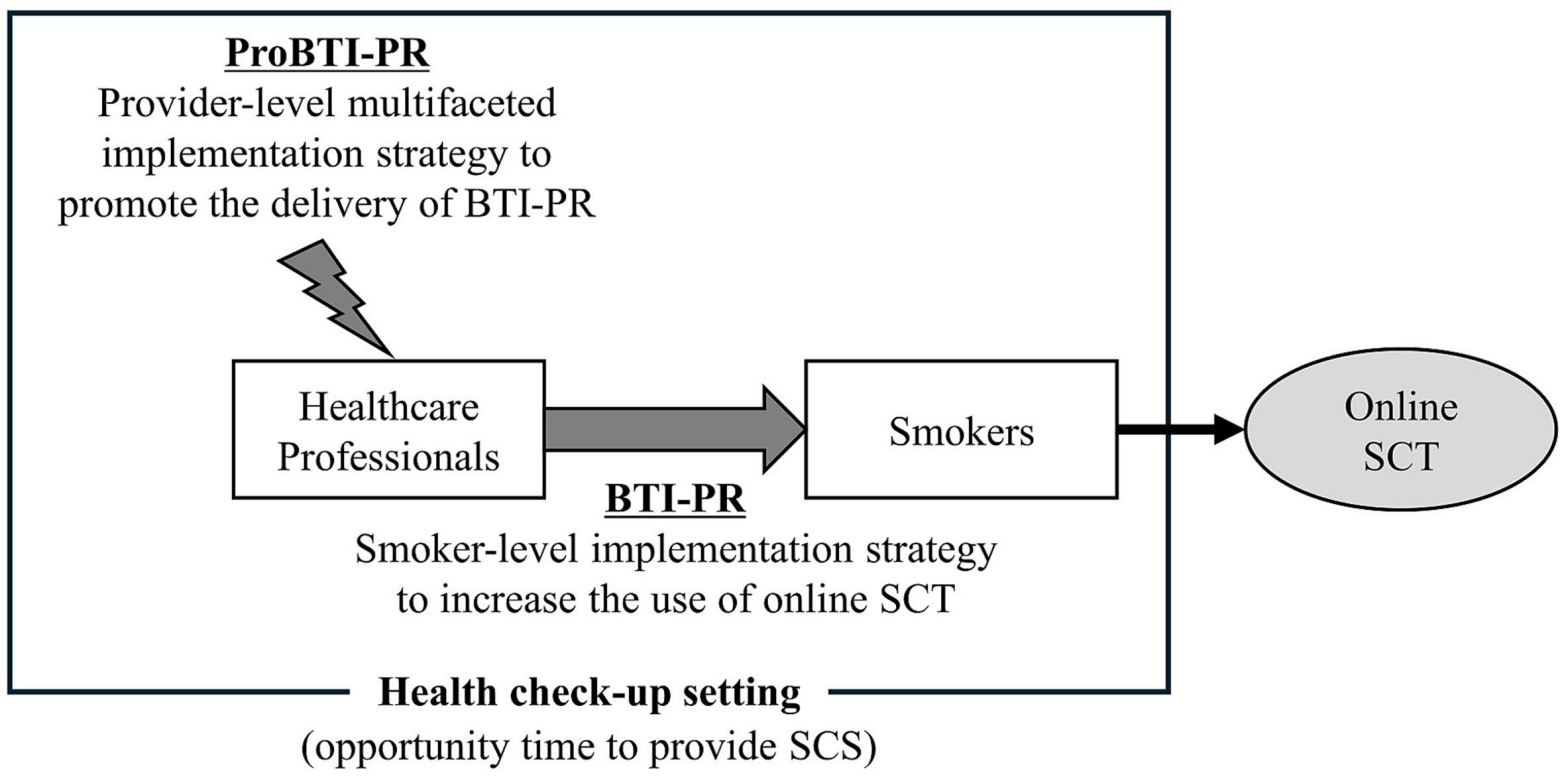
Overview of BTI-PR and ProBTI-PR. BTI-PR, Brief Tobacco Intervention with Proactive Referral; ProBTI-PR, a multifaceted provider-level implementation strategy to promote delivery; SCT, smoking cessation treatment.

This study is a single-arm, multi-center, uncontrolled hybrid type 3 effectiveness-implementation study (24–26) to estimate smoker uptake of SCT following the ProBTI-PR, a multifaceted provider-level implementation strategy to promote delivery of the BTI-PR to smokers in health check-up settings (Figure 1). The BTI-PR is a smoker-level implementation strategy to increase the use of online SCT. A logic model of the BTI-PR and ProBTI-PR is shown in Supplementary Figure 1. It incorporates individual factors to influence behavior (Determinants), methods for influencing positive change in the determinants (Change Methods), and a specific technique for practical use of the theoretical change method (Practical Application) (27). Change Methods are described using the taxonomy proposed by Kok et al., a theory-based method to influence behaviors through changing determinants (28). Detailed procedures of the BTI-PR and ProBTI-PR are described in the following sections. A primary outcome is the penetration of the online SCT, which is defined as the proportion of smokers eligible for the BTI-PR who use the online SCT. In this study, smokers are referred by the BTI-PR to a fully online SCT to eliminate geographical limitations on access to in-person SCT and to enable measurement of the primary outcome.

### Smoker-level implementation strategy: BTI-PR

The BTI-PR is an implementation strategy that aims to motivate smokers to use online SCT. In a previous study, the BTI-PR was developed by adapting proactive referral to health check-up settings (23). In the first step of the adaptation process, researchers (KY, TS) developed a prototype of the adapted proactive referral based on the findings of previous studies on barriers to implementing SCS in health check-up settings (22) and proactive referrals in clinic or community settings (13, 29–33). The researchers then consulted with experts on smoking cessation support (CT, MN) and the management staff of a health check-up center (KK) to confirm the BTI-PR.

**Figure 2:**
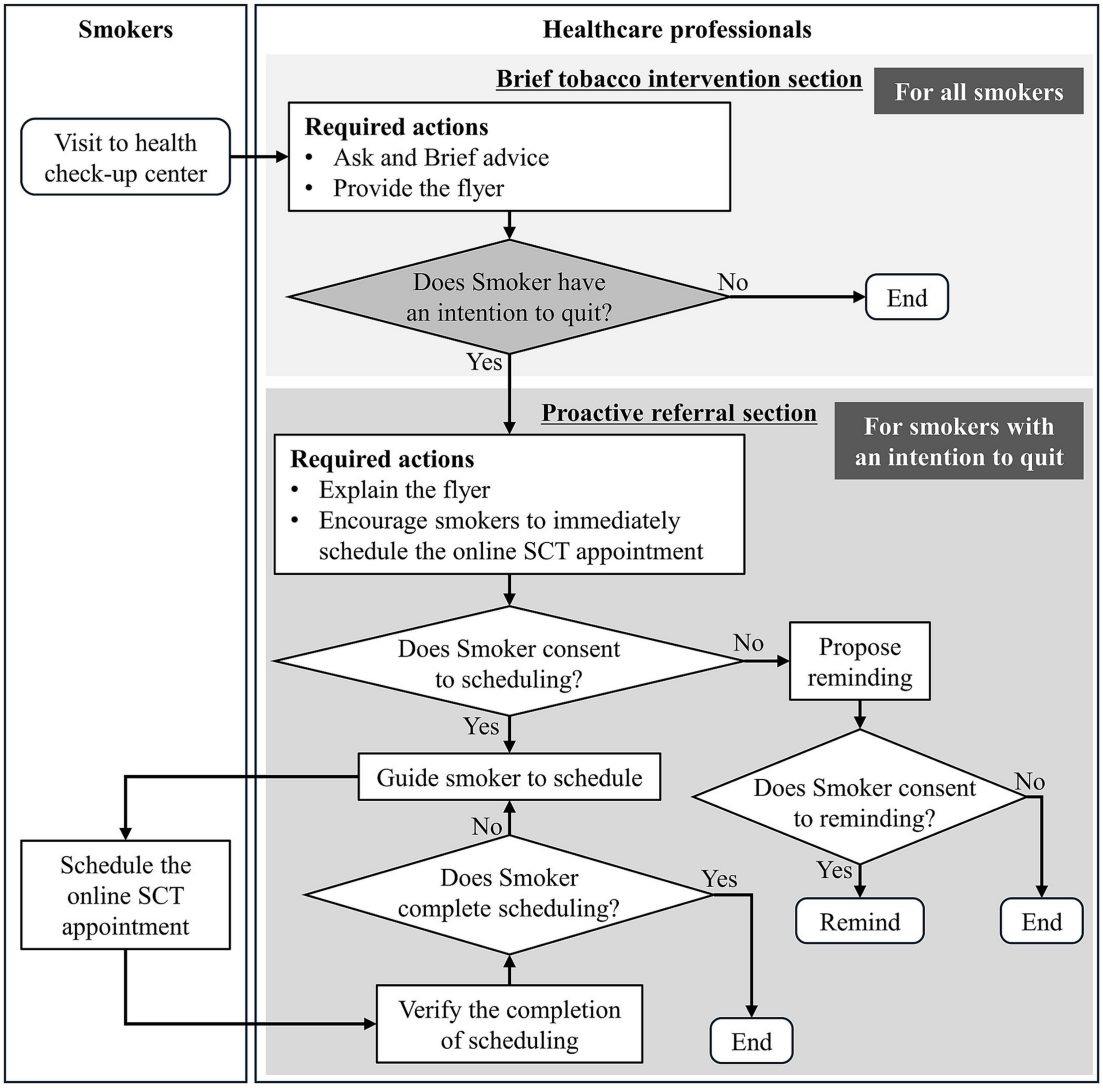
Flow of BTI-PR. BTI-PR, Brief Tobacco Intervention with Proactive Referral; SCT, smoking cessation treatment.

The BTI-PR integrates proactive referral into the ABR method, a brief SCS recommended for healthcare professionals working in time-constrained settings in Japan, including health check-up settings. The ABR method is composed of three steps – “Ask,” “Brief advice,” and “Refer” (19). After first asking the smoker about their tobacco use and intention to quit smoking as “Ask,” the professional then explains the importance of quitting and suggests solutions for quitting as “Brief advice.” Finally, the professional refers smokers expressing an intention to quit to cessation resources, including SCT, as “Refer.” The BTI-PR is segmented into a brief tobacco intervention section and a proactive referral section (Figure 2). BTI-PR elements specified by Proctor’s Reporting Framework (34) and their core functions, defined as the core purpose of each element in these strategies (35, 36), are described in Table 1. Provision of the BTI-PR is expected to take less than 5 min.

**Table 1:** Specification of the smoker-level implementation strategy (BTI-PR).

**Element**	**Core functions**	**Action**	**Target** **individuals**	**Temporality**	**Dose**	**Implementation outcome affected**	**Justification**
Brief tobacco intervention section							
(A) Ask and Brief advice	Identify smokers with the intention to quit and motivate them to quit	Ask smokers about their tobacco use and their intention to quit, record smoker’s intention to quit, and advise smokers to quit by explaining the importance of quitting and suggesting solutions for quitting	All smokers	Beginning of the brief tobacco intervention section	Once	Fidelity of the BTI-PR, penetration of SCT	Manual for smoking cessation support
(B-1) Provide the flyer	Provide information on the online SCT for smokers	Provide smokers with a flyer, including information on the online SCT	All smokers	At the same time as Brief advice	Once	Penetration of SCT	Manual for smoking cessation support
Proactive referral section							
(B-2) Explain the flyer	Provide information on the online SCT for smokers	Explain the contents of the flyer	Smokers with the intention to quit smoking	After the brief tobacco intervention section	Once	Penetration of SCT	Manual for smoking cessation support
(C-1) Encourage to schedule the online SCT	Propose smokers to schedule an online SCT appointment at the health check-up center	Encourage smokers to immediately schedule an appointment for the online SCT and record whether smokers consent to scheduling	Smokers with the intention to quit smoking	After explaining the flyer	Once	Penetration of SCT	Proactive referral
(C-2) Guide to schedule the online SCT	Support smokers to schedule smoothly	Guide smokers to schedule the appointment by themselves during their stay at the health check-up center	Smokers who consent to scheduling	After obtaining the smoker’s consent to the appointment	At least once until completion	Penetration of SCT	Proactive referral
(D-1) Propose to remind	Maintain a connection with smokers	Propose reminding smokers about scheduling the appointment and record whether smokers consent to be reminded	Smokers who do not consent to scheduling	After confirming whether smokers give consent to scheduling	Once	Penetration of SCT	Proactive referral
(D-2) Remind	Motivate smokers to schedule the online SCT appointment	Remind smokers about scheduling	Smokers who consent to the reminder	Within a week after the health check-up	Once	Penetration of SCT	Proactive referral

BTI-PR, Brief Tobacco Intervention with Proactive Referral; SCT, smoking cessation treatment.

In the brief tobacco intervention section, healthcare professionals ask all smokers about their tobacco use and intention to quit (Ask), and then advise them to quit (Brief advice). Further, healthcare professionals provide smokers with a flyer during the Brief advice which includes information on the efficacy of SCT, the cost and advantages of online SCT compared to in-person SCT, and instructions on how to schedule and receive the online SCT. “Ask and Brief advice” and “Provide the flyer” are defined as required actions for all smokers.

The proactive referral section is designed for smokers who have an intention to quit smoking. Therefore, smoker’s intention to quit is a main decision point of the BTI-PR. After explaining the contents of the flyer, the professional then encourages the smoker to immediately schedule an online SCT appointment. “Explain the flyer” and “Encourage smokers to immediately schedule the online SCT appointment” are defined as required actions for smokers with an intention to quit smoking. The professional confirms the smoker’s consent to scheduling this appointment. For smokers who consent to scheduling, the professional guides them to schedule the appointment by themselves and verifies their completion of the appointment process. If a smoker cannot complete the appointment process, the professional provides additional support, such as suggesting alternative means of scheduling, and subsequently verifies completion again. For smokers who do not consent to scheduling, the professional proposes to the smoker that they remind them about scheduling the appointment later, after they leave the health check-up center; if the smoker consents to being reminded, the professional discusses with the smoker the optimal means and timing of the reminder. At the predetermined time, which is required to be within a week after the health check-up, the professional reminds the smoker to schedule the online SCT appointment once.

### Provider-level multifaceted implementation strategy: ProBTI-PR

The ProBTI-PR is a multifaceted implementation strategy designed to facilitate healthcare professionals working in health check-up centers to deliver the BTI-PR. The ProBTI-PR consists of a bundle of eight strategies: (a) conduct a training session on SCS, (b) provide a guide on the BTI-PR, (c) provide a checksheet for the BTI-PR, (d) provide a flyer on the online SCT, (e) conduct a kickoff meeting and request the discussion of pre-specifiable matters, (f) schedule a pre-implementation phase, (g) request holding a review meeting and report, and (h) conduct a plenary meeting to share local knowledge. Based on the findings of the previous feasibility study (23), strategies (a), (b), (c), (d), and (e) have been improved, while (f), (g) and (h) have been newly added. Each strategy of the ProBTI-PR is specified based on Proctor’s Reporting Framework (34) in Table 2, including core functions (35, 36) and categories classified by the Expert Recommendations for Implementing Change (ERIC) (37). In addition, strategies are justified by previous studies (23, 38, 39) and constructs of the Consolidated Framework for Implementation Research (CFIR) (40).

**Table 2:** Specification of the provider-level multifaceted implementation strategy (ProBTI-PR).

**Implementation strategy**	**ERIC classification**	**Core functions**	**Actor and action**	**Temporality**	**Dose**	**Implementation outcome affected**	**Justification**
(a) Conduct a training session on SCS	Develop educational materials Make training dynamic	(1) Increase provider’s knowledge on SCS, the harm of smoking, and the benefits of quitting.	(1) Researchers arrange an online self-education program for providers.	Preparation phase	Once (1 h)	Penetration, fidelity, implementation cost	Provider’s knowledge, attitude, self-efficacy, and behaviors related to SCS improved significantly following completion of the online self-education program.
		(2) Increase provider’s skill and self-efficacy in delivering the SCS.	(2) Researchers provide a lecture, and facilitate the discussion in a practical exercise using simulation cases.	Preparation phase (after the self-education)	Once (in-person lecture [1 h] and practical exercise [1.5 h])		The group work was developed based on the findings of a previous study showing that participants in the education program had an increased frequency of SCS delivery.
(b) Provide a guide to the BTI-PR	Develop educational materials	Enhance the provider’s understanding of the BTI-PR.	Researchers provide paper-based materials describing the objectives and workflow of the BTI-PR.	Preparation phase (in the kickoff meeting)	One set of materials for each provider	Penetration, fidelity, acceptability, appropriateness, feasibility	A guide outlining procedures step-by-step enhances understanding of BTI-PR delivery, based on cognitive load theory.
(c) Provide a checksheet for the BTI-PR	Remind clinicians	Enable the provider to deliver the BTI-PR smoothly and ensure its quality	Researchers provide paper-based materials outlining the steps of the BTI-PR.	Preparation phase (in the kickoff meeting)	One set of materials for each smoker	Penetration, fidelity, acceptability, appropriateness, feasibility	Confirmation of BTI-PR procedures on-site facilitates establishment of the habit of BTI-PR delivery.
(d) Provide a flyer on the online SCT	Prepare patients/consumers to be active participants	Facilitate the dissemination of information on the online SCT and smoking cessation to smokers.	Researchers provide a flyer explaining the online SCT and smoking cessation.	Preparation phase (in the kickoff meeting)	One set of materials for each smoker	Penetration, fidelity	A flyer on the online SCT facilitates establishment of the habit of BTI-PR delivery.
(e) Conduct a kickoff meeting and request the discussion of pre-specifiable matters	Conduct educational meetings	(1) Facilitate provider’s familiarity with the BTI-PR.	Researchers distribute the materials and explain details of the BTI-PR.	Preparation phase (after the training session)	Once (30 min)	Penetration, fidelity, implementation cost, acceptability, appropriateness, feasibility	A kickoff meeting enhances understanding of BTI-PR delivery, based on cognitive load theory.
		(2) Tailor the BTI-PR to each health check-up center	Healthcare professionals discuss and report about pre-specifiable matters	Preparation phase (after the training session)	Once		Adaptation of the BTI-PR to each health check-up center improves its feasibility and enhances self-efficacy.
(f) Schedule a pre-implementation phase	Stage implementation scale up	Build practical experience with the BTI-PR to increase its skill and self-efficacy	Healthcare professionals deliver the BTI-PR to at least five smokers	At the start of the BTI-PR delivery	2 months	Penetration, fidelity, feasibility	Findings of a feasibility study indicated that self-efficacy for BTI-PR delivery increased after practical experience with it.
(g) Request holding a review meeting and report	Conduct local consensus discussions	Tailor the BTI-PR to each health check-up center based on practical experience with the BTI-PR	Healthcare professionals discuss and report the adaptation of the BTI-PR	Pre-implementation phase	At least once	Penetration, fidelity, implementation cost, acceptability, appropriateness, feasibility	Adaptation of the BTI-PR based on practical experience improves its feasibility and enhances self-efficacy.
(h) Conduct a plenary meeting to share local knowledge	Capture and share local knowledge	Share the experience and adaptation of the BTI-PR	Researchers conduct the meeting with the participation of healthcare professionals	Pre-implementation phase	Once (1 h)	Penetration, implementation cost	Sharing local knowledge functions as vicarious experience within the framework of social cognitive theory, thereby enhancing self-efficacy.

BTI-PR, Brief Tobacco Intervention with Proactive Referral; CFIR, Consolidated Framework for Implementation Research; ERIC, Expert Recommendations for Implementing Change; ProBTI-PR, a multifaceted provider-level implementation strategy to promote delivery of the BTI-PR in health check-up settings; SCS, smoking cessation support; SCT, smoking cessation treatment. Dose includes both frequency and duration.

(a) Conduct a training session on SCS

The 3.5-h training session aims to help healthcare professionals acquire the knowledge and skills required to deliver SCS. This session is composed of an online self-education program and a group workshop. Initially, healthcare professionals receive the online self-education program using Japan Smoking cessation Training Outreach Project (J-STOP) Next (41), an e-learning program, to improve the providers’ knowledge of SCS (38). To facilitate completion of the self-education program within 1 h, an expert on SCS (CT) selected video lectures and quizzes with explanations of SCS in health check-up settings, the harm of smoking, and the benefits of quitting from among the web-based contents provided on J-STOP Next. The number of items in this self-education program has been reduced from that in the previous feasibility study because the participants of that study reported that they were unable to complete the original program within the intended one-hour timeframe.

Following the self-education, healthcare professionals participate in the group workshop. This active learning session was designed based on the findings of a previous study of an educational program on SCS for providers and aims to increase provider’s skill and self-efficacy in delivering the SCS (39). The group workshop consists of a 1-h in-person lecture and a 1.5-h practical exercise. In the in-person lecture, the expert provides a lecture on nicotine dependency and SCS. In the practical exercise, healthcare professionals discuss how to advise smokers to increase the importance and self-efficacy of smoking cessation using simulation cases. The expert facilitates this discussion.

(b) Provide a guide to the BTI-PR

To improve understanding of BTI-PR delivery, a paper-based guide is provided to healthcare professionals. The guide outlines the BTI-PR procedure step-by-step, based on the cognitive load theory (42). The guide provides an overview of the BTI-PR, including its objective and workflow, and also reflects modifications made to other strategies based on the previous feasibility study.

(c) Provide a checksheet for the BTI-PR

To enable healthcare professionals to deliver the BTI-PR smoothly and ensure its quality, a paper-based checksheet outlining the BTI-PR procedure is provided to them. Healthcare professionals can confirm BTI-PR procedures on-site, which facilitates the establishment of a habit of BTI-PR delivery. To allow healthcare professionals to customize the checksheet in accordance with conditions at their particular health check-up center, researchers have newly clarified the following four modifiable items as pre-specifiable matters: (1) personal information of smokers to be recorded in the checksheet (e.g., telephone numbers or birthdays); (2) phrases to use to encourage smokers to schedule online SCT appointments and to propose the reminder to them; (3) devices to use to schedule an online SCT appointment (e.g., smokers’ smartphones or center-provided computers); and (4) methods to use for the reminder (e.g., short message services or telephone calls). This modification of pre-specifiable matters does not disturb core functions of the BTI-PR, as described in Table 1. Healthcare professionals will use one checksheet for each smoker to record the provider’s actions and the smoker’s responses. Healthcare professionals who deliver the BTI-PR record whether they deliver each required action, including “Ask and Brief advice,” “Provide the flyer,” “Explain the flyer”, and “Encourage smokers to immediately schedule the online SCT appointment”, using a checksheet with preidentified checkboxes. When healthcare professionals deliver each action, they mark the corresponding checkbox during delivery of the BTI-PR. Other provider’s actions, including the verification of appointment completion, proposal for a reminder, determination of the means and timing of the reminder, conduct of the reminder, and time spent on BTI-PR delivery, are also recorded in the checksheet. Smoker responses recorded in the checksheet include their intention to quit and the status of consent to the scheduling of the online SCT and reminder.

(d) Provide a flyer on the online SCT

To facilitate the dissemination of information on online SCT to smokers, healthcare professionals are provided with a flyer. The flyer was originally developed for the feasibility study and contains information on the efficacy and cost of online SCT, the advantages of online SCT compared to in-person SCT, and instructions on how to schedule an online SCT appointment and receive the online SCT. It has been updated to include information on the treatment plan of the online SCT and, to create an image of quitting among smokers, the physical improvement felt by ex-smokers and ex-smokers’ opinions. Healthcare professionals distribute the flyer to all smokers and explain its contents to those expressing an intention to quit.

(e) Conduct a kickoff meeting and request the discussion of pre-specifiable matters

Subsequent to the training session, a 30-min kickoff meeting is held in-person or online. The BTI-PR procedure is explained step-by-step, based on cognitive load theory (42). In this meeting, researchers provide healthcare professionals with an explanation of the BTI-PR’s flow, as outlined in the guide, and some advice on how to use the checksheet and the flyer.

After the kickoff meeting, healthcare professionals are newly requested to adapt the BTI-PR to their center by discussing and determining the optimal timing for implementing the BTI-PR within the check-up flow at each health check-up center, as well as the pre-specifiable matters. This adaptation of the BTI-PR is expected to improve its feasibility and enhance self-efficacy for its delivery among healthcare professionals. Healthcare professionals report the optimal timing of the BTI-PR, pre-specifiable matters, and the time spent on preparation for BTI-PR delivery, including discussion, to researchers as a kickoff report via an online form. Moreover, healthcare professionals modify the checksheet in accordance with the pre- specifiable matters and send it to the researchers via e-mail.

(f) Schedule a pre-implementation phase

The two-month period following the preparation phase, in which the training session and kickoff meeting take place, is designated as the pre-implementation phase. The findings of the feasibility study indicated that self-efficacy for the BTI-PR among healthcare professionals gradually increased not only after the training session but also with practical experience with the BTI-PR (23). In the pre-implementation phase, each healthcare professional is recommended to deliver the BTI-PR to no fewer than five smokers in order to build practical experience with it.

(g) Request holding a review meeting and report

Healthcare professionals are requested to conduct at least one review meeting at each health check-up center during the pre-implementation phase. The purpose of this meeting is to discuss the adaptation of the BTI-PR to their health check-up center, based on their practical experience with the BTI-PR. The adaptation of the BTI-PR is expected to improve its feasibility and enhance self-efficacy in delivery among healthcare professionals. Healthcare professionals report the meeting proceedings, including the meeting date, to researchers as a review report via an online form. Furthermore, they send a modified checksheet to researchers via e-mail if they modify the checksheet.

(h) Conduct a plenary meeting to share local knowledge

In the plenary meeting, healthcare professionals from all health check-up centers participate and share their experience and adaptation of the BTI-PR under the guidance of researchers. Sharing local knowledge functions as vicarious experience within the framework of social cognitive theory (43), and thereby enhances self-efficacy for BTI-PR delivery. This 1-h meeting is conducted in person or online during the pre-implementation phase.

### Study setting

This study is conducted in eight health check-up centers of the Niigata Association of Occupational Health (NAOH) in Niigata Prefecture, Japan. Six of these centers (Group 1) are ready to receive the ProBTI-PR immediately, while the remaining two (Group 2) are unable to do so at this time. Therefore, the penetration of the online SCT following the ProBTI-PR is estimated in Group 1. The health check-up centers in Group 1 are located across both urban and rural areas. The modified ProBTI-PR, which is adapted to low-resource settings based on the findings in Group 1, is then provided to Group 2. No comparison between Group 1 and Group 2 is planned. Findings of post-adaptation from Group 2 will be reported in a future paper.

NAOH has provided health check-ups as well as both in-person and online SCT. The online SCT to which smokers are referred in this study is provided by NAOH. In this online SCT, consisting of five online sessions within 12 weeks in accordance with the standard procedure for SCT in Japan (5), smokers receive behavioral counseling remotely and are sent medications for nicotine replacement therapy. The online SCT is not currently covered by public health insurance, and costs 45,000 Japanese yen (approximately 300 US dollars). In contrast, smokers pay 15,000 yen (approximately 100 US dollars) for insurance-covered in-person SCT. However, given expectations that the online SCT will be covered by public health insurance in the future (44), the difference in cost (30,000 yen) is covered by research funding to anticipate this potential future implementation setting. Consequently, the out-of-pocket cost for smokers who use the online SCT in this study will be 15,000 yen.

### Participants and recruitment

The participants of this study consist of providers who are the target of the ProBTI-PR, and smokers who receive the BTI-PR. Inclusion criteria for healthcare professionals are as follows:

1) Employment at one of eight health check-up centers of the NAOH.
2) Certified public health nurse, registered nurse, or registered dietitian.
3) In charge of SCS at health check-ups.
4) Consent to participation in the study.

Eligible providers will receive adequate written and oral explanation of the study details, including its procedures, potential risks, and benefits prior to enrollment.

Inclusion criteria for smokers eligible for the BTI-PR (eligible smokers) are as follows:

1) Age 20 years or older.
2) Insured by the Japan Health Insurance Association (JHIA), the largest health insurance association for workers employed in small or medium-sized companies and their dependents in Japan.
3) Visit to one of eight health check-up centers within the study period.
4) Self-identified as a current smoker in the questionnaire for the health check-up.

The requirement to obtain consent from smokers is waived for the following three reasons, as specified in ethical guidelines (45): (1) the BTI-PR and data collection procedures are deemed to pose no more than minimal risk; (2) the waiver will not have any negative consequences on the rights and welfare of smokers; and (3) obtaining consent from smokers will be infeasible in the practice of health check-up centers and will negatively affect generalizability because smokers without an intention to quit smoking are expected to decline consent. All smokers are provided with a document which describes the study details and are provided the opportunity to refuse the use of their data in this study.

### Study design

This study is planned as a single-arm, uncontrolled study to evaluate the penetration of online SCT through the BTI-PR delivered by healthcare professionals who receive the ProBTI-PR among Group 1. The primary aim of this study is to estimate penetration following the ProBTI-PR under real-world conditions, rather than to test comparative effectiveness. Given this implementation-focused objective, a concurrent control group is expected to provide limited incremental information for penetration. Our previous study revealed that fewer than 0.2% of smokers used SCT within 3 months after their health check-up (22). Moreover, between June 2022 and July 2023, no smokers used the online SCT provided by NAOH, which was covered by public health insurance. Given this consistently negligible baseline uptake under usual SCS, evaluating absolute penetration following implementation was considered more informative than conducting a comparative effectiveness assessment. In addition, there are practical constraints on center-level randomization. Specifically, only six of the eight centers are ready to implement the ProBTI-PR and could be included in a cluster randomization scheme. Given this small number of clusters, randomization alone would be unlikely to ensure adequate comparability between groups. Therefore, a single-arm design was considered appropriate for achieving the study objective. While the primary outcome is defined for Group 1, the same variable is also concurrently measured in Group 2 to describe penetration of the online SCT within the usual SCS.

### Study timeline

**Figure 3:**
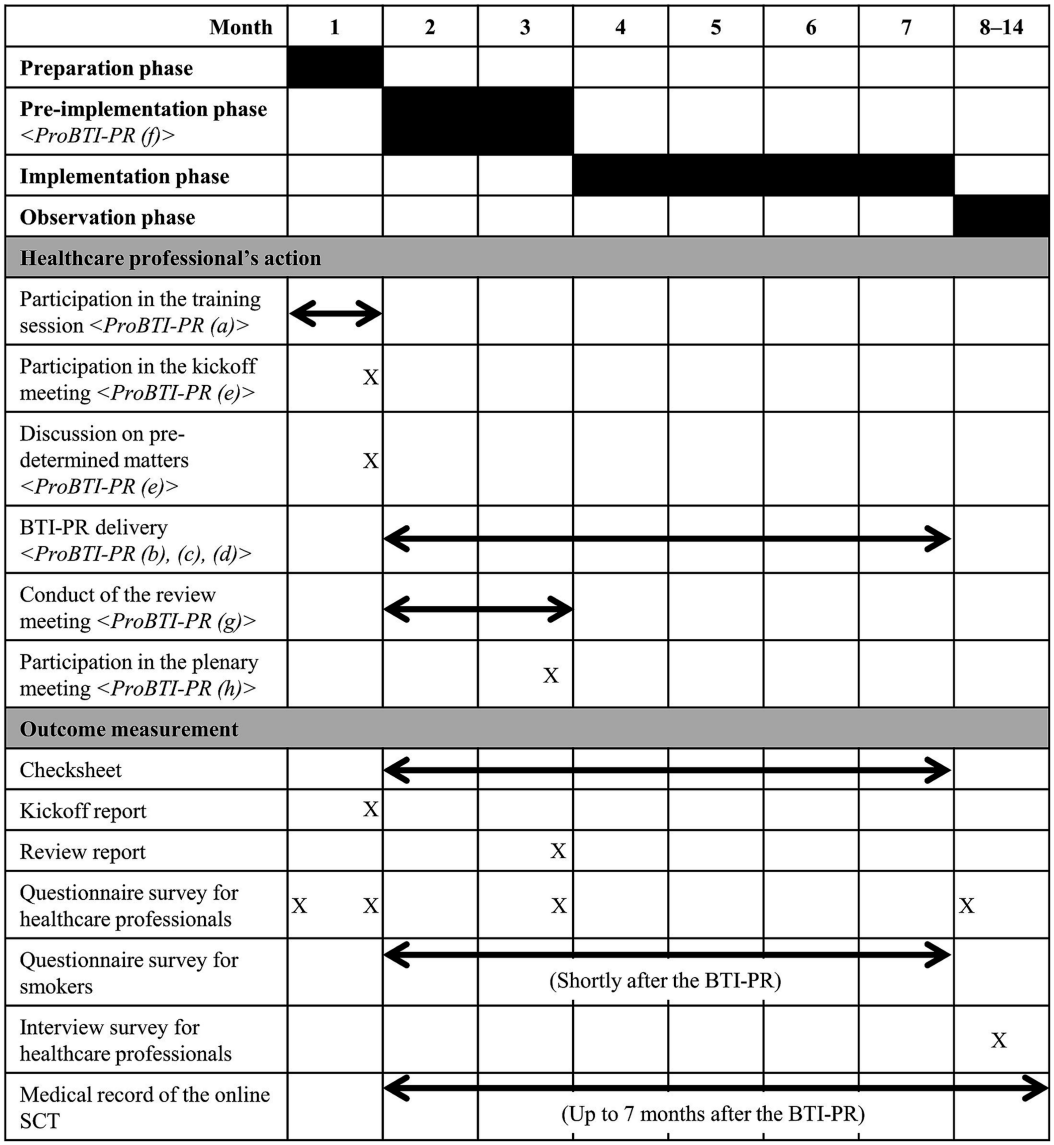
Timeline of this study. BTI-PR, Brief Tobacco Intervention with Proactive Referral; ProBTI-PR, a multifaceted provider-level implementation strategy to promote delivery; SCT, smoking cessation treatment.

This study is divided into four phases (Figure 3): a preparation phase (in January 2025), a pre-implementation phase (from February to March 2025), an implementation phase (from April to July 2025), and an observation phase (from August 2025 to February 2026). First, healthcare professionals attend the training session and the kickoff meeting during the one-month preparation phase. They also discuss pre-specifiable matters in this phase. Subsequently, during the 2-month pre-implementation phase, healthcare professionals commence delivery of the BTI-PR and discuss their adaptation of the BTI-PR in the review meeting. Then, during the implementation phase, healthcare professionals deliver the adapted BTI-PR for four months. After the end of the implementation phase, an observation phase for outcomes related to SCT use will continue for up to 7 months. Moreover, a revised version of the ProBTI-PR based on findings among Group 1 will then be provided to healthcare professionals in Group 2.

### Data collection

Data will be collected from the checksheet, the kickoff and review reports, questionnaire surveys for healthcare professionals and smokers, the interview survey for healthcare professionals, the health check-up record, and the medical record of the online SCT (Tables 3, 4). The questionnaire survey for healthcare professionals is conducted four times online: at the beginning of the preparation phase (Survey 1), the end of the preparation phase (Survey 2), the end of the pre-implementation phase (Survey 3), and after the implementation phase (Survey 4). These surveys include the following questions: baseline characteristics (Survey 1); frequency of SCS delivery in the health check-up setting (39, 46) (Surveys 1, 3, and 4); self-efficacy, value, importance and normalization of SCS delivery (39, 46–48) (Surveys 1 to 4); acceptability, appropriateness, and feasibility of BTI-PR delivery (49) (Survey 2 to 4); and time spent on self-education and satisfaction with the training session (Survey 2). In the questionnaire survey for smokers, which is conducted shortly after BTI-PR delivery, smokers are asked about the acceptability and appropriateness of receiving the BTI-PR (49), and smoking cessation measures implemented at their worksite. The interview survey for healthcare professionals is conducted on a health check-up center basis online. The objective of this semi-structured interview survey is to identify determinants of the implementation and sustainment of the BTI-PR. The interview guide was developed based on the Capability, Opportunity, and Motivation for Behavior (COM-B) model (50) and the Theoretical Domains Framework (TDF) (51). Questions and the interview guide for these surveys are described in the Supplementary File.

**Table 3:** Provider-level outcomes and data source.

Outcome	Definition	Data source							
		Checksheet	Kickoff report	Review report	Questionnaire survey for healthcare professionals				Interview survey
					1	2	3	4	
Outcomes related to SCS delivery									
Frequency of SCS delivery	Mean score (0 to 4)				X		X	X	
Self-efficacy of SCS delivery	Mean score (0 to 10)				X	X	X	X	
Value of SCS delivery	Mean score (0 to 10)				X	X	X	X	
Importance of SCS delivery	Mean score (0 to 10)				X	X	X	X	
Normalization of SCS delivery	Mean score (0 to 10)				X	X	X	X	
Outcomes related to BTI-PR delivery									
Fidelity of BTI-PR delivery	Proportion of smokers who are delivered all required actions according to the smoker’s intention to quit smoking	X							
Introduction cost for BTI-PR delivery	Mean of the total time for the preparation of the BTI-PR delivery multiplied by the per-hour salary		X	X		X			
Delivery cost of the BTI-PR	Mean time for the BTI-PR delivery multiplied by the per-hour salary	X							
Acceptability of BTI-PR delivery	Mean score (0 to 4)					X	X	X	
Appropriateness of BTI-PR delivery	Mean score (0 to 4)					X	X	X	
Feasibility of BTI-PR delivery	Mean score (0 to 4)					X	X	X	
Determinants for BTI-PR delivery	Qualitative description								X
**Others**									
Satisfaction with the training session	Mean score (0 to 4)					X			

BTI-PR, Brief Tobacco Intervention with Proactive Referral; SCS, smoking cessation support. Time points of questionnaire survey: survey 1, at the beginning of the preparation phase; survey 2, at the end of the preparation phase; survey 3, at the end of the pre-implementation phase; survey 4, after the implementation phase. Required actions according to the smoker’s intention to quit smoking: “Ask and Brief advice” and “Provide the flyer” for all smokers, “Explain the flyer” and “Encourage smokers to immediately schedule the online SCT appointment” for smokers with an intention to quit smoking.

**Table 4:** Smoker-level outcomes and data sources.

Outcome	Definition	Data source			
		Health check-up record	Medical record	Checksheet	Questionnaire survey for smokers
Outcomes related to the BTI-PR					
Consent to scheduling the online SCT	Proportion of smokers who consent to scheduling the online SCT among smokers with intention to quit			X	
Consent to being reminded	Proportion of smokers who consent to being reminded among smokers who do not consent to scheduling			X	
Acceptability of receiving the BTI-PR	Proportion of smokers who accept the BTI-PR				X
Appropriateness of receiving the BTI-PR	Proportion of smokers who perceive the BTI-PR as appropriate				X
Outcomes related to the online SCT					
Appointment at the health check-up center	Proportion of smokers who schedule the online SCT at the health check-up center among eligible smokers	X		X	
Appointment within 1 month	Proportion of smokers who schedule the online SCT within 1 month after the BTI-PR among eligible smokers	X	X		
Penetration of the online SCT*	Proportion of smokers who attend the first session of the online SCT within 3 months after the BTI-PR among eligible smokers	X	X		
Completion of the online SCT	Proportion of smokers who complete the online SCT among smokers who use the online SCT		X		
Outcomes related to health behavior					
Achievement of smoking cessation	Proportion of smokers who achieve self-reported point-prevalence abstinence at the last session of the online SCT among eligible smokers	X	X		

BTI-PR, Brief Tobacco Intervention with Proactive Referral; SCT, smoking cessation treatment. * Primary outcome.

### Outcome

The primary outcome is penetration of the online SCT among smokers eligible for the BTI-PR, i.e., all smokers who are satisfied with the inclusion criteria, during the implementation phase. Penetration is one of the implementation outcomes, and is used as an indicator of service utilization or delivery (52). Penetration of the online SCT is defined as the proportion of eligible smokers who attend the first session of the online SCT within 3 months after receiving the BTI-PR; or is in other words an indicator of service utilization. Smokers who do not meet the criteria for the SCT covered by public health insurance (5) are included in eligible smokers (denominator of penetration) but excluded from smokers who use the online SCT (numerator of penetration), in anticipation of a future setting in which online SCT is covered by public health insurance. The criteria to receive the SCT under public health insurance are as follows: intention to quit smoking immediately; diagnosis of nicotine dependence (Tobacco Dependence Screener score ≥5 points) (53); Brinkman index ≥200; and signing of a consent document for SCT. Smokers who use other smoking cessation services, such as in-person SCT and over-the-counter smoking cessation medications purchased at pharmacies, are not captured. Penetration of the online SCT is also measured among smokers who receive the BTI-PR during the pre-implementation phase and smokers who receive the usual SCS in Group 2.

The provider-level secondary outcomes encompass items related to SCS delivery, namely frequency, self-efficacy, value, importance, and normalization; those related to BTI-PR delivery, namely, fidelity, implementation costs (introduction and delivery costs), acceptability, appropriateness, feasibility, and determinants; and satisfaction with the training session. The smoker-level secondary outcomes encompass items related to the BTI-PR, namely consent to scheduling the online SCT and being reminded, and acceptability and appropriateness of the BTI-PR; the online SCT, namely appointment (at the health check-up center and within 1 month after health check-up) and completion; and health behavior, namely achievement of smoking cessation (i.e., effectiveness outcome). Achievement of smoking cessation is assessed as self-reported point-prevalence abstinence at the last session of the online SCT. Determinants of BTI-PR delivery are qualitative outcomes, while the others are quantitative outcomes. Definitions of these outcomes are described in Tables 3, 4.

### Statistical analysis

The baseline characteristics of both the healthcare professionals and smokers are summarized with descriptive statistics. Data from health check-up records, namely smokers’ age and gender, are mandatory items in health check-ups and missing values are not expected. Penetration of the online SCT, i.e., the primary outcome, is calculated by dividing the number of smokers who attend the first session of the online SCT within 3 months after the BTI-PR, as determined by the medical record of the online SCT, by the number of eligible smokers, as determined by the health check-up record. The penetration of the online SCT corresponds to an individual-level population-average proportion. Because attendance at the first session of the online SCT is ascertained from medical records, the primary outcome data are expected to be available for all eligible smokers. Moreover, because linkage among health check-up records, checksheets, and medical records of online SCT is conducted using unique identifiers, linkage failure is not expected. Given the single-arm design, the interpretation of findings will focus on whether the pre-specified threshold is achieved rather than on causal inference. Based on the findings of previous studies and the consensus among stakeholders, it is hypothesized that penetration of the online SCT will be 5%. Previous studies of clinical settings indicated that the penetration of smoking cessation services increased from 0.15–2.0% with usual SCS to 1.6–14.7% with proactive referral (13, 54–59). Furthermore, should penetration reach 5% or higher through provision of the ProBTI-PR, decision-makers from the JHIA and NAOH have agreed to the introduction and sustainment of the BTI-PR in health check-up settings. Therefore, the 5% threshold was pragmatically determined as an implementation-focused benchmark. The ProBTI-PR is considered effective if the lower limit of its 95% confidence interval is 2% or higher. To account for potential within-cluster correlation, 95% confidence intervals for the penetration of the online SCT are calculated using a modified Wilson score interval method for clustered binary outcome data, which incorporates the intracluster correlation coefficient (ICC) into the variance formula (60). Clustering is considered at the health check-up center level. Center-specific results will also be presented descriptively to allow assessment of potential between-center heterogeneity. Regarding the secondary provider-level quantitative outcomes, proportions or mean scores and 95% confidence intervals are calculated using the same method as for the primary outcome. In addition, penetration of the online SCT is stratified by smoker’s intention to quit and by health check-up center.

The fidelity of BTI-PR delivery is defined as the proportion of eligible smokers who receive the BTI-PR from healthcare professionals as intended, namely the delivery of all required actions according to the smoker’s intention to quit smoking. Fidelity is calculated by dividing the number of smokers who are delivered all required actions, as determined by the checksheet, by the number of eligible smokers. The action is counted as not delivered if the checkbox is not marked. In addition, the proportion of smokers receiving each required action is evaluated as partial fidelity. Furthermore, fidelity is stratified by health check-up center. The introduction cost for BTI-PR delivery is calculated by the total time for preparation of BTI-PR delivery, including the training session and several meetings, multiplied by the per-hour salary of healthcare professionals. Delivery cost of the BTI-PR is calculated by the mean time required for delivery, which is determined using the record in the checksheet, multiplied by the per-hour salary of healthcare professionals. The mean time required for delivery of the BTI-PR will be calculated using available data only. The per-hour salary of healthcare professionals is estimated by the latest Basic Survey on Wage Structure (61). Other provider-level secondary outcomes derived from responses to the questionnaire survey for healthcare professionals, including the frequency of SCS delivery and acceptability of BTI-PR, will be analyzed using available data only. Achievement of smoking cessation is defined as the proportion of eligible smokers who report smoking cessation at the last session of the online SCT. Missing smoking status data at the last session of online SCT are treated as smoking cessation not achieved. Other smoker-level secondary outcomes derived from responses to the questionnaire survey for smokers, namely the acceptability and appropriateness of BTI-PR, will be analyzed using available data only. The statistical analysis application R (62) is used for these analyses.

Qualitative outcomes, i.e., determinants of BTI-PR delivery, are explored through deductive coding of interview transcripts according to the corresponding domains of the COM-B model (50) and the TDF (51) assigned to each interview question.

### Sample size calculation

To account for within-cluster correlation in a cluster-level intervention, ICC has been incorporated into the sample size calculation (63). A previous study showed that 21.4% of smokers were referred to the Quitline through SCS and a fax referral delivered by clinic staff, and the ICC was reported as 0.024 (64). Another study showed that the proportion of smokers who achieved smoking cessation through SCS delivered by counselors in community settings was 18.4%, and the ICC was reported as 0.015 (65). Consequently, the ICC was assumed to be 0.02 in this study. Assuming a 5% penetration rate for the online SCT, a sample size of 215 smokers was estimated to achieve a two-sided 95% confidence interval with a width of 6% under an individual-level intervention without clustering. To estimate the design effect for cluster-level intervention, the number of eligible smokers was estimated using the actual number of eligible smokers who visited health check-up centers in 2023, assuming a 15% decrease in attendance. The expected number of eligible smokers during 4 months from April to July was 817. Using this expected number of eligible smokers and the assumed ICC (0.02), the design effect was calculated to be 3.70. Accordingly, the required sample size for cluster-level intervention was calculated as 798 smokers, which was lower than the expected number of eligible smokers (n = 817). Therefore, we decided on an implementation phase of 4 months, from April to July 2025. The timing of this period was pragmatically determined based on the limited availability of healthcare professionals to participate in the training sessions.

### Adaptation of the ProBTI-PR to low-resource settings

To enhance the feasibility of the BTI-PR in diverse health check-up settings, including those with limited resources, we plan to further adapt the ProBTI-PR based on the study findings in Group 1 and explore provider fidelity to the adapted BTI-PR in Group 2. For instance, human resource limitations may hinder organization of the group workshop of the training session by SCS experts.

## Results

Although 25 healthcare professionals in Group 1 initially consented to participate in the study, one withdrew due to reassignment to other duties prior to the implementation phase. Consequently, 24 healthcare professionals who received the ProBTI-PR delivered the BTI-PR to smokers in the implementation phase. Data collection for these healthcare professionals and smokers in Group 1 was completed in February 2026, and the final analysis for outcomes was commenced in March 2026. We anticipate that a 5% penetration of the online SCT will facilitate stakeholders in adopting the BTI-PR and ProBTI-PR in health check-up settings.

## Discussion

In this paper, we describe the study method for evaluating the implementation-focused effectiveness of ProBTI-PR, a multifaceted provider-level implementation strategy to promote delivery of the adapted proactive referral in health check-up settings, i.e., BTI-PR. To our knowledge, this is the first study to determine the penetration of online SCT among smokers who receive a proactive referral in health check-up settings. The penetration estimated in this study may reflect not only the BTI-PR together with the ProBTI-PR but also other factors, including healthcare professional-related factors (e.g., readiness to adopt the BTI-PR and increased attention to the delivery of SCS due to study participation), smoker-related factors (e.g., secular trends of intention to quit smoking and familiarity with telemedicine), and online SCT-related factors (e.g., availability of online SCT and reduced out-of-pocket costs). Therefore, this study is not intended to isolate the contribution of the independent effect of the ProBTI-PR alone to penetration. Moreover, studies on implementation strategies that facilitate the delivery of SCS by healthcare professionals working in health check-up settings are scarce. The implementation process of the BTI-PR will be examined by measuring several implementation outcomes (52), including fidelity and implementation cost. Acceptability, appropriateness, and feasibility of the BTI-PR and determinants of its delivery will also be evaluated through questionnaire and interview surveys for healthcare professionals, given that implementation of the BTI-PR during routine health check-ups may increase their workload. In the feasibility study, the mean time required for delivery of the BTI-PR was 3.7 min per smoker (23). A similar amount of time is expected to be required in this study, which may increase the workload associated with routine health check-ups.

The findings of this study, conducted in health check-up settings where healthcare professionals can reach large numbers of smokers, will provide relevant stakeholders, including insurers of public health insurance and health policymakers, with evidence necessary to support the implementation of the BTI-PR, together with the ProBTI-PR. Accordingly, the findings will be widely disseminated not only through publication of peer-reviewed international scientific journals but also through communication with relevant stakeholders. In addition, the findings may provide insights into the implementation of proactive referral to countries or regions where health check-up systems are well established.

In this study, both the BTI-PR and ProBTI-PR are specified using a reporting framework, which will enhance the reproducibility of implementation strategies in future research and practice (34). Core functions of the ProBTI-PR are also specified in Table 2. These core functions should be preserved during adaptation of the ProBTI-PR to low-resource settings. For example, although the group workshop should include active learning to increase healthcare professionals’ skill and self-efficacy in delivering the SCS, which is a core function of the workshop, its form, such as in-person or web-based session, can be adapted according to available resources.

Nevertheless, several limitations should be acknowledged. First, this study is designed as a single-arm study, which precludes comparison of outcomes with and without the ProBTI-PR. Therefore, this study cannot estimate the causal effect of the ProBTI-PR on penetration of online SCT. Consequently, the findings of this study may be influenced by several factors other than the ProBTI-PR. However, the primary aim of this study is to estimate penetration following the ProBTI-PR under real-world conditions in order to provide stakeholders with the implementation-focused evidence necessary to support decision-making regarding the adoption of the BTI-PR together with the ProBTI-PR, which can be evaluated using a single-arm study design. Second, this study is designed on the assumption that online SCT will in future be covered by public health insurance. Therefore, research funding is used to subsidize the cost of online SCT so that smoker’s out-of-pocket expenses are equivalent to those for in-person SCT. If such insurance coverage is not realized, the findings of this study may not be fully generalizable. Nevertheless, the findings may still be informative in settings where the cost of online SCT is covered as part of a workplace health promotion program. Third, smoking cessation services other than online SCT are not captured in this study. Therefore, overall uptake of smoking cessation service following ProBTI-PR may be underestimated. Fourth, several outcomes, including the fidelity and acceptability of the BTI-PR, are assessed by self-reported measures, which may introduce social desirability bias (66). For example, fidelity is assessed based on provider self-reporting without independent observation, audit, or validation. Therefore, fidelity may be subject to social desirability bias or incomplete recording. To reduce such bias, the questionnaire survey employs validated scales where available. Fifth, determinants influencing the use of online SCT among smokers, including digital literacy and accessibility of in-person SCT, will not be directly assessed due to the practical challenges of recruiting eligible smokers during health check-ups. In particular, although online SCT may reduce geographic barriers to receiving SCT, it may also introduce digital access barriers. Moreover, smokers with easy access to in-person SCT may perceive less benefit from online SCT. These determinants will instead be explored indirectly through the interview survey with healthcare professionals. Sixth, caution is needed when generalizing the findings to unemployed individuals and workers in large companies because their characteristics may differ from those of workers in small- and medium-sized companies, of whom the participants are representative. In addition, the findings may not be directly generalizable to smokers who do not receive health check-ups.

## Conclusion

This study is expected to provide relevant stakeholders with the evidence necessary to support the adoption of the adapted proactive referral together with its implementation strategy in health check-up settings, albeit that the single-arm design does not allow causal inferences regarding effectiveness. The findings of this study will contribute to the implementation of effective SCS, including proactive referral, into preventive health services.

## Data Availability

The datasets presented in this article are not readily available because the security provisions specified in the protocol and as approved by the institutional review board but are available from the corresponding author on reasonable request. Requests to access the datasets should be directed to TS, tshimazu@ncc.go.jp.
