# Supplementary material for: Study method for a single-arm, multi-center implementation study of strategies for use by healthcare professionals in offering smokers an adapted proactive referral for online smoking cessation treatment in health check-up settings: a hybrid type 3 effectiveness-implementation study (N-EQUITY2405)
Source: Front Public Health. 2026 Jul 15;14:1843479. doi: 10.3389/fpubh.2026.1843479 (PMC13414892; doi:10.3389/fpubh.2026.1843479)
Supplement: Supplementary file 1 [file Data_Sheet_1.docx]

**Supplementary file**

**Supplementary Figure**

**
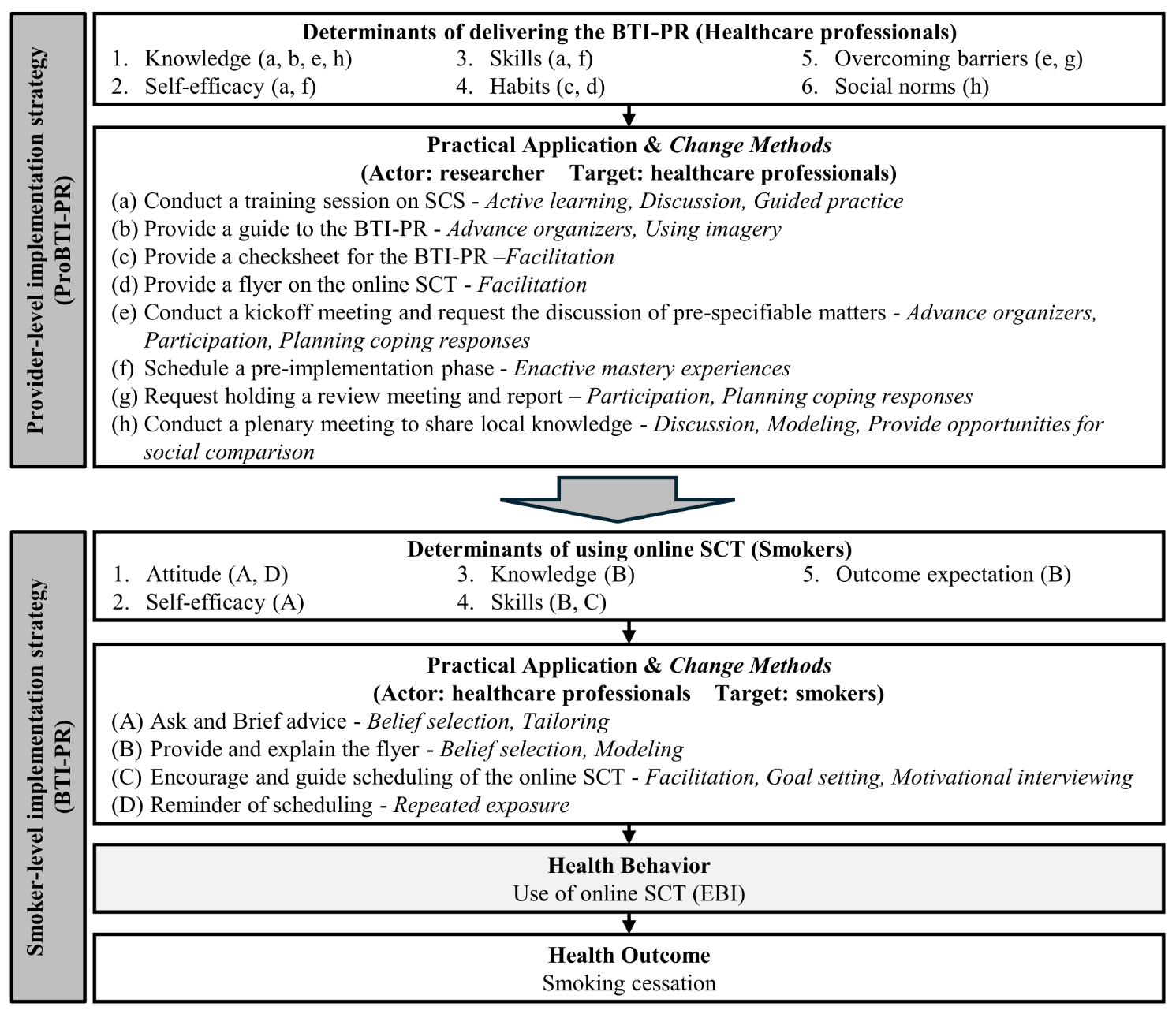
**

Supplementary Figure 1. Logic model of BTI-PR and ProBTI-PR

BTI-PR, brief tobacco intervention with proactive referral; EBI, evidence-based intervention; ProBTI-PR, a multifaceted provider-level implementation strategy to promote delivery of the BTI-PR in health check-up settings; SCS, smoking cessation support; SCT, smoking cessation treatment

**Questionnaire survey for healthcare professionals**

The questionnaire survey for healthcare professionals is conducted four times online: at the beginning of the preparation phase (Survey 1), at the end of the preparation phase (Survey 2), at the end of the pre-implementation phase (Survey 3), and after the implementation phase (Survey 4). The following questions are combined to formulate the questionnaire at each use. The survey design ensures that no missing data occurs, except for the open-text field on the training session.

1. **Baseline characteristics (Survey 1)**

Please tell us about yourself.

1. Please select your gender.

🞎 Female

🞎 Male

1. Please select the number of years you have worked in health guidance during health checkups (if you have previously worked at other health check-up centers, include these periods as well).

🞎 Less than 1 year

🞎 1-2 years

🞎 3-5 years

🞎 6-10 years

🞎 11-15 years

🞎 16 years or more

1. Please select all of your medical qualifications.

🞎 Public health nurse

🞎 Registered nurse

🞎 Registered dietitian

1. **Frequency of SCS delivery in the health check-up setting ^1 2^ (Surveys 1, 3, and 4)**

We would like to ask you about the smoking cessation support that you provide during health check-ups. To what extent have you done the following?

1. Ask about the tobacco use status of health check-up recipients.

🞎 Always 🞎 Usually 🞎 Sometimes 🞎 Rarely 🞎 Never

1. Advise smokers receiving a health check-up to quit smoking.

🞎 Always 🞎 Usually 🞎 Sometimes 🞎 Rarely 🞎 Never

1. Assess the willingness of smokers receiving a health check-up to quit smoking (identify their stage).

🞎 Always 🞎 Usually 🞎 Sometimes 🞎 Rarely 🞎 Never

1. Provide smokers receiving a health check-up with specific guidance on how to quit.

🞎 Always 🞎 Usually 🞎 Sometimes 🞎 Rarely 🞎 Never

1. Introduce smoking cessation treatment, etc., to smokers receiving a health check-up.

🞎 Always 🞎 Usually 🞎 Sometimes 🞎 Rarely 🞎 Never

1. **Self-efficacy (Question 1), value (Question 2), importance (Question 3), and normalization (Question 4-6) of SCS delivery ^1-4^ (Surveys 1 to 4)**

Please rate your feelings about smoking cessation support during health check-ups on a scale from 0 to 10. Select the number that best applies to you.

1. How confident are you in providing smoking cessation support to smokers receiving health check-ups?

(0 = Not at all confident, 5= Somewhat confident, 10 = Completely confident)

0 1 2 3 4 5 6 7 8 9 10

1. Do you feel that smoking cessation support is valuable during health check-ups?

(0 = Not at all valuable, 5= Somewhat valuable, 10 = Very valuable)

0 1 2 3 4 5 6 7 8 9 10

1. Do you feel that quitting smoking is important for smokers receiving health check-ups?

(0 = Not at all important, 5= Somewhat important, 10 = Very important)

0 1 2 3 4 5 6 7 8 9 10

1. When you deliver smoking cessation support, how familiar does it feel?

(0 = Still feels very new, 5= Feels somewhat familiar, 10 = Feels completely familiar)

0 1 2 3 4 5 6 7 8 9 10

1. Do you feel that smoking cessation support is currently a normal part of your work in health check-ups?

(0 = Not at all, 5= Somewhat, 10 = Completely)

0 1 2 3 4 5 6 7 8 9 10

1. Do you feel that smoking cessation support will become a normal part of your work in health check-ups?

(0 = Not at all, 5= Somewhat, 10 = Completely)

0 1 2 3 4 5 6 7 8 9 10

1. **Acceptability (Question 1), appropriateness (Question 2), and feasibility (Question 3) of BTI-PR delivery ^5^ (Surveys 2 to 4)**

We would like to ask your feelings about the BTI-PR. To what extent do you agree with the following statements?

1. I approve of the delivery of the BTI-PR as health guidance in health check-up settings.

🞎 Completely agree 🞎 Agree 🞎 Neither agree nor disagree

🞎 Disagree 🞎 Completely disagree

If you select “Disagree” or “Completely disagree”, please write the reasons for your selection.

1. It seems suitable for smokers to receive delivery of the BTI-PR as a health guidance in health check-up settings.

🞎 Completely agree 🞎 Agree 🞎 Neither agree nor disagree

🞎 Disagree 🞎 Completely disagree

If you select “Disagree” or “Completely disagree”, please write down reasons.

1. The BTI-PR seems implementable as a component of health guidance in health check-up settings.

🞎 Completely agree 🞎 Agree 🞎 Neither agree nor disagree

🞎 Disagree 🞎 Completely disagree

If you select “Disagree” or “Completely disagree”, please write the reasons for your selection.

1. **Time spent on the self-education (Question 3) and satisfaction (Question 1, 2, 4) with the training session (Survey 2)**

We would like to ask you about the training session.

1. How satisfied were you with the training session overall?

🞎 Very satisfied 🞎 Somewhat satisfied 🞎 Neither satisfied nor dissatisfied

🞎 Somewhat dissatisfied 🞎 Completely dissatisfied

1. How satisfied were you with the online self-education program as part of the training session?

🞎 Very satisfied 🞎 Somewhat satisfied 🞎 Neither satisfied nor dissatisfied

🞎 Somewhat dissatisfied 🞎 Completely dissatisfied

🞎 I don’t know because I have not completed the online self-education program yet

1. The self-education program was expected to take 60 minutes to complete as part of the training session. Please select how much time you actually spent on the self-education program.

🞎 60 minutes or less

🞎 61 to 90 minutes

🞎 91 to 120 minutes

🞎 121 to 150 minutes

🞎 151 to 180 minutes

🞎 181 minutes or more

1. How satisfied were you with the group workshop as part of the training session?

🞎 Very satisfied 🞎 Somewhat satisfied 🞎 Neither satisfied nor dissatisfied

🞎 Somewhat dissatisfied 🞎 Completely dissatisfied

🞎 I don’t know because I didn’t take the group workshop

1. Please write any comments you may have about the training session.

**Questionnaire survey for smokers**

Smokers are asked about the acceptability (Q1-2) and appropriateness (Q1-3) of receiving the BTI-PR ^5^, and smoking cessation measures implemented in their worksite (Q2). This survey is conducted shortly after BTI-PR delivery.

Q1-1. Did you receive an explanation about scheduling an appointment for smoking cessation treatment during this health check-up?

🞎 Yes 🞎 No (skip to Q2)

Q1-2. Did you welcome receiving the explanation about the appointment for smoking cessation treatment at this health check-up?

🞎 Completely welcomed it 🞎 Welcomed it 🞎 Neither welcomed nor did not welcome it

🞎 Unwelcomed it 🞎 Completely unwelcomed it

Q1-3. Do you agree that it is suitable for smokers to be given an explanation about making an appointment for smoking cessation treatment during the health check-up?

🞎 Completely agree 🞎 Agree 🞎 Neither agree nor disagree

🞎 Disagree 🞎 Completely disagree

Q2. Please select all smoking cessation measures implemented in your workplace.

🞎 No smoking on the premises or indoors

🞎 Installation of designated smoking areas

🞎 Prohibition of smoking during working hours

🞎 Permission to receive smoking cessation treatment during work hours

🞎 Recommendation for taking leave to receive smoking cessation treatment

🞎 Assistance for smoking cessation treatment cost

🞎 Provision of nicotine replacement therapy (patches and gum) or assistance with its cost

🞎 Awards and cash rewards for those who successfully quit smoking

🞎 Non-smoker allowance

🞎 Seminar on the health effects of smoking

🞎 Professional smoking cessation support for smokers

🞎 Others ( )

🞎 No implementation of smoking measures

**Interview guide for healthcare professionals**

Hello. My name is [Interviewer Name] from the National Cancer Center. Thank you for taking the time to meet with us today despite your busy schedule. The interview is scheduled to last 60 minutes. We look forward to talking with you.

<Explanation of this interview study>

Prior to the interview, we would like to confirm a few things. This interview survey was added to the study after you were recruited to it.

- In this interview, we will ask you about your thoughts on the Brief Tobacco Intervention with Proactive Referral (BTI-PR). Our aim is to explore factors influencing the delivery of the BTI-PR and to determine effective strategies for increasing and sustaining its delivery.
- We will record the interview in order to transcribe and review its content.
- The information collected will be stored by the principal investigator within the National Cancer Center under strict security and will be used by researchers involved in this study.
- The information collected will be used in reports for the Ministry of Health, Labour and Welfare's grant, as well as in academic presentations and papers, but only in an aggregated form that does not identify you by name.
- You may withdraw from the interview at any time during or after the interview for any reason. In such cases, you will not suffer any disadvantage. In addition, you will not be criticized for your answers.

Do you have any questions or concerns about the interview?

Do you consent to participate in the interview survey?

(If no) That’s all for today. Thank you very much.

(If yes) Let's begin the interview.

[Start recording here]

<Interview>

This interview is divided into six parts: (1) knowledge and skills on smoking cessation support, (2) confidence in BTI-PR delivery, (3) thoughts based on experience with BTI-PR delivery, (4) relationships with smokers during BTI-PR delivery, (5) relationships with colleagues and supervisors during BTI-PR delivery, and (6) the sustainability of BTI-PR delivery.

First, we would like to ask you about knowledge and skills in smoking cessation support.

- What knowledge and skills did you gain about smoking cessation support by participating in this study?
- What was most helpful in gaining that knowledge and skills?
- Is there any other knowledge or skills you would like to gain? What do you think would be necessary to gain them?

Second, we would like to ask you about confidence in BTI-PR delivery.

- Did you become more confident in BTI-PR delivery by participating in this study?
- (If yes) What made you feel more confident?
- (If no) Why didn’t you feel more confident?
- What would help you feel more confident about delivering the BTI-PR?

Third, we would like to ask you about your thoughts based on experience with BTI-PR delivery.

- Which factors had the greatest impact on implementing the BTI-PR? For example, what made it easier or more difficult to implement?
- Did implementing the BTI-PR have any advantages or disadvantages for you?
- Did you have any concerns when implementing the BTI-PR? What were they?
- How did implementing the BTI-PR affect your regular work? This includes both positive and negative impacts.
- What ideas or rules did you create to implement the BTI-PR within the health check-up process?
- What resources, such as personnel, materials, or time, are lacking for implementation of the BTI-PR?

Fourth, we would like to ask you about your relationship with smokers during BTI-PR delivery.

- How did delivering the BTI-PR affect your relationship with smokers?
- How did the reactions of smokers who received the BTI-PR affect your subsequent BTI-PR delivery?

Fifth, we would like to ask you about your relationships with colleagues and supervisors during BTI-PR delivery.

- How did you collaborate with your colleagues to deliver the BTI-PR?
- Did your supervisor help you with BTI-PR delivery? What kind of support did you receive?
- What kind of support from your supervisor would be helpful for continuing delivery of the BTI-PR?
- Did you have an opportunity to share information about the BTI-PR with other facilities outside of the plenary meeting?

Finally, we would like to ask you about the sustainability of BTI-PR delivery.

- As part of your daily work at the health check-up center, would you like to continue delivering the BTI-PR? Do you feel able to do that?
- What factors would help you to continue or increase delivery of the BTI-PR within the health guidance you provide during check-ups?

It's about time to wrap up today's interview. Is there anything else you'd like to share or ask?

Based on the valuable feedback we received today, we will consider making improvements to the BTI-PR. Thank you again for your time today.

**Reference**

1. Taniguchi C, Sezai I, Yoshimi I, et al. Effectiveness of a smoking cessation educational program for Japanese nurses on subsequent changes of behavior in delivering smoking cessation counseling. *Tob Induc Dis* 2022;20(February):1-9. doi: 10.18332/tid/144649

2. Sezai I, Taniguchi C, Yoshimi I, et al. How Self-Efficacy toward, Perceived Importance of, and Beliefs about Smoking Cessation Support Impact-Related Behaviors in Japanese Nursing Professionals. *Int J Environ Res Public Health* 2022;19(4):2304.

3. Borrelli B, Lee C, Novak S. Is provider training effective? Changes in attitudes towards smoking cessation counseling and counseling behaviors of home health care nurses. *Prev Med* 2008;46(4):358-63. doi: https://doi.org/10.1016/j.ypmed.2007.09.001

4. Finch TL, Girling M, May CR, et al. Improving the normalization of complex interventions: part 2 - validation of the NoMAD instrument for assessing implementation work based on normalization process theory (NPT). *BMC Med Res Methodol* 2018;18(1):135. doi: 10.1186/s12874-018-0591-x

5. Weiner BJ, Lewis CC, Stanick C, et al. Psychometric assessment of three newly developed implementation outcome measures. *Implementation Science* 2017;12(1):108. doi: 10.1186/s13012-017-0635-3
